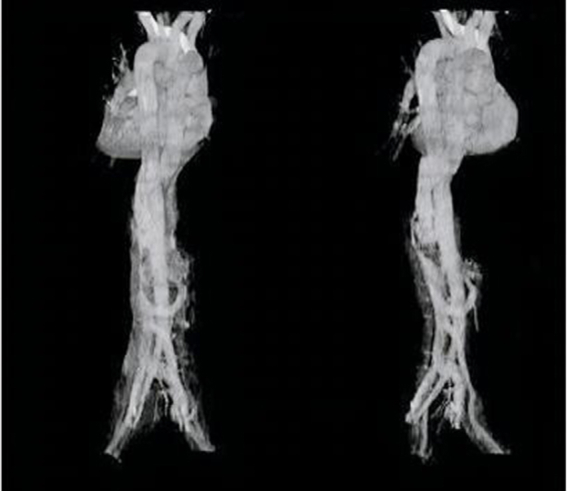# Exploring isolated inferior vena cava anomalies beyond the norm

**DOI:** 10.1016/j.jvsv.2025.102306

**Published:** 2025-08-29

**Authors:** Micaela R. Cuneo, Diane F. Hale

**Affiliations:** Department of General Surgery, Brooke Army Medical Center, Fort Sam Houston, TX

Anomalies of the inferior vena cava (IVC) are typically caused by abnormal embryologic development.^1^ These can be discovered after patients display symptoms of venous insufficiency and congestion, but some are discovered incidentally. In young patients with an unprovoked deep vein thrombosis (DVT), anomalies of the IVC are important to consider and rule out, as they are implicated in up to 5% of patients younger than 30 years with idiopathic unprovoked DVTs.^2^

**Figure:**
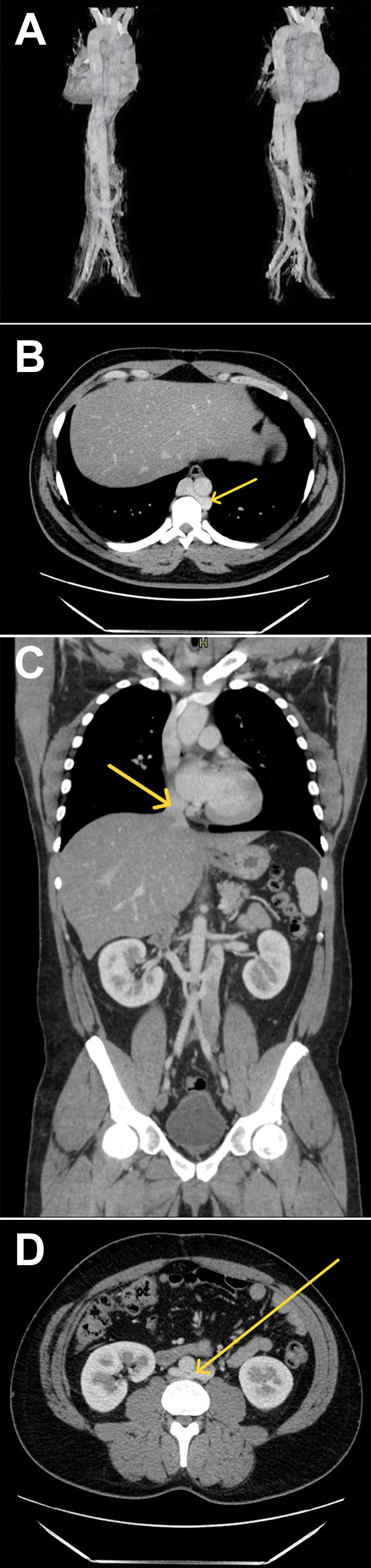

Abnormal IVC development typically occurs in the sixth week of embryogenesis as primitive veins form and regress. This can also lead to anomalous venous return of the intra-abdominal organs.^1^^,^^3^ The true incidence of isolated IVC abnormalities is unclear because some patients, such as this one, may be asymptomatic.

## Case report

The patient is a 37-year-old man with a past medical history of hepatic steatosis who presented to clinic for removal of an axillary soft tissue mass. He consents to publication. He has a history of a childhood operation to remove a “cyst” in his right abdomen, but he cannot recall that procedure and does not have those records. A chest computed tomography (CT) was obtained to characterize the mass, and the patient was incidentally found to have an azygos continuation of the IVC. He denies any history of DVT, lower extremity edema, or venous stasis ulcers.

A venous phase CT was obtained to further characterize his vascular anatomy. This revealed a distal azygos continuation (*A*/Cover and *B*) as well as hepatic venous drainage into a short segment IVC draining to the right atrium (*C*). There was also a retroaortic right renal vein identified (*D*). Since discovery of this incidental IVC anomaly, he has not developed any thromboses or peripheral venous disease.

## Discussion

Azygos continuation of the IVC is a rare presentation of congenital venous abnormality. There are several reports in the literature of deep venous abnormalities that are found incidentally as part of workup for another condition. In one study of 7972 patients who underwent CT for other reasons, 12 (0.15%) were found to have an IVC anomaly. As imaging has become more readily available, these anomalies are identified more frequently in asymptomatic patients.^4^

## Funding

None.

## Disclosures

None.

## Figures and Tables

**Cover image:**